# The use of mouthguards and related factors among basketball players in Indonesia

**DOI:** 10.1186/s12903-023-03480-3

**Published:** 2023-11-04

**Authors:** Herry Novrinda, Putri Tianda Lambe, Risqa Rina Darwita, Jae-Young Lee

**Affiliations:** 1 Department of Dental Public Health and Preventive Dentistry, Universitas Indonesia, Jakarta, Indonesia; 2 Faculty of Dentistry, Universitas Indonesia, Jakarta, Indonesia; 3 Department of Dental Hygiene, College of Health Science, Dankook University, Chungnam, Korea

**Keywords:** Mouthguards, Injury, Social support, Sport dentistry, Structural equation model

## Abstract

**Background:**

Basketball is a sport with a higher injury rate. Regardless, few basketball players use mouthguards, which predisposes them to injuries. The use of mouthguards (UoM) could be related to several factors. This study aims to identify factors associated with UoM and to construct a model from the factors among basketball players in Indonesia.

**Methods:**

Through convenience sampling, a total of 286 among basketball players in Indonesia was included in this cross-sectional study. These participants filled out online a modified questionnaires regarding demographic and several factors related to UoM. The data was analyzed using chi-square test, independent-sample t-test, binary logistic regression, and structural equation modeling (SEM).

**Results:**

There were 286 players. 127 of them were males and 159 were females. Of them, 86 (30.1%) used mouthguards. Age, duration (in year), and weekly practicing basketball (in hour) were all significantly different between mouthguards users and non-users with (*p* = 0.005, *p* = 0.036 and *p* = 0.035), respectively. The UoM was significantly associated with level of awareness, injury experience, social support, and oral health professional (OHP) support with (*p* = 0.002, *p* < 0.001, *p* < 0.001 and *p* < 0.001), respectively. This result was also supported by a variety of variables’ ORs, which range from 1.28 to 5.97. The coefficient of determination (R^2^) was 0.27.

**Conclusions:**

The UoM among basketball players in Indonesia was related to several factors, including the level of knowledge, level of awareness, duration of basketball career, injury experiences, social support, and oral health professionals’ support which was constructed to propose a model. The model could explain 27% of the relationship between variables and UoM among Indonesian basketball players. This model will be useful for more comprehensive initiatives to promote oral health. It might be applicable for other countries as well as other sports communities / physical activities.

## Introduction

Due to the rising prevalence of health conditions like temporomandibular joint disorders, orofacial injuries, dental caries and erosion, periodontal disease, and defective occlusion, athletes’ oral health is now receiving a lot of attention on a global scale [1]. Dental injury is also a major public health issue due to its prevalence in society and the wide-ranging impact it can have on a person’s quality of life [2]. Sports, especially those that involve physical contact (such as basketball), are associated with a high risk of injury to the teeth and mouth [3]. In a previous study, it was found that between group sports and contact sports, basketball has the greatest risk of orofacial injury [4]. These types of injuries are commonly related to several factors, including the awareness of players, personal experience, the interventions of oral health professionals, and the surrounding environment [3] as well as insufficient awareness among athletes and the general public about the prevention of dental and orofacial injuries [5]. Orofacial injuries sustained while participating in sports are largely avoidable. A mouthguard or mouth protection is one method of dental prevention against orofacial injuries [5] which has been shown to reduce the risk of sports-related dental injuries [6]. It is a piece of equipment that covers the teeth and surrounding mucosa in order to prevent or reduce trauma to the teeth, gingival tissues, lips, and jaws [7]. Prefabricated mouthguards (stock mouthguards that are not fitted to the patient); mouth-formed or “boil-and-bite” mouthguards (made from a thermoplastic material that softens and molds when heated); and custom-made (fabricated from dental impressions) mouthguards are available nowadays. Among them, the customized mouthguard is the best and offers the most protection. Furthermore, it interferes with oral functions less and is the most commonly recommended by dentists [8–11]. However, athletes frequently do not use a mouthguard despite being aware of its protective capability; thus, they have been found to have poor compliance with mouthguard use even after having sustained previous oral-facial injuries [9]. Furthermore, several previous studies on the use of mouthguards (UoM) and related factors from various countries have been published, and a number of factors such as age [12], gender, knowledge, awareness, time spent playing basketball, athlete attitude, injury experience [12] [13], as well as social influences by the media, coaches, teammates and family are linked to the use of mouthguards [14–17].

Basketball is one of the most high-ranking (sixth out of nine) popular sports in Indonesia [18]. Along with the growing public interest in basketball, several dental and orofacial injuries occur, necessitating preventive action on the part of athletes. However, with such interest in basketball and the effectiveness of mouthguards, to our knowledge, there is no study on the UoM and related factors among basketball players in Indonesia as well as the first study which proposed a model using structural equation modelling (SEM). A proposed model that encompasses various factors is needed to either prevent or manage an injury or disease more comprehensively. Hence, the aims of this study were (1) to identify the factors associated with the UoM among basketball players in Indonesia and (2) to propose a model from various related factors associated with the UoM using SEM. The proposed modelling would be constructed from a combination of several variables that first showed meaningful findings in the descriptive and bivariate analysis (such as the t-test, chi-square, and logistic regression).

Based on the findings and proposed factors from previous mentioned studies [12–17], The study’s null hypotheses were (1) the level of knowledge (LoK), injury experience, awareness, duration of basketball career, support from the social environment and oral health professionals, were identified and associated significantly with the UoM among basketball players in Indonesia, (2) based on SEM analysis, these factors could be directly or indirectly connected significantly or insignificantly to construct a model with a high coefficient of determinants.

## Materials and methods

### Study design and sample

This cross-sectional study was conducted from November to December of the year 2021 via convenience sampling among basketball players in Indonesia. This study was approved by The Faculty of Dentistry Universitas Indonesia Research Ethics Committee (approval no.66/Ethical Approval/FKGUI/IX/2021), and electronic informed consent (via Google form) was obtained from each participant. This study was conducted per the principles of the World Medical Association Declaration of Helsinki [19] and Ethical Standards in Sport and Exercise Science Research [20]. The online *Raosoft calculator* (http://www.raosoft.com/samplesize.html) was used to estimate the sample size. This strategy had been performed in previous studies [21, 22]. According to the previous studies, the power of the test was set at 95% with a 5% error margin and a response distribution of 20% (assuming that the proportion of players used the mouthguards in another previous study [23]), resulting in a minimum sample size of 243 players. To allow for participant drop-out, a total of 286 players was included in this study.

### Data collection

Participants were contacted through a network of basketball players. A total of 286 basketball players from several provinces in Indonesia (categorized into Java islands and outside Java) were willing to participate. Since this study was conducted during the COVID-19 pandemic, the questionnaire (in Google forms) was distributed through WhatsApp and other social media platforms. These questionnaires also contained information regarding this study, encompassing the background, aim, confidentiality, etc., as well as a willingness statement.

### Research instruments

The research instrument was a modified questionnaire from a previous study entitled “Basketball players’ experience of dental injury and awareness about mouthguards in China” [16]. To achieve our study objectives, it was translated into Indonesian and paraphrased as well. The questionnaire was then translated back into English by three researchers in order to accomplish the goal of our study. The modified questionnaire encompassed respondents’ attributes, including name, email address, age, sex, each one’s origin location, height, weight, basketball career duration (in years), number of hours spent playing basketball per week, knowledge and awareness regarding dental injury and mouthguards, dental injury experience, social support, and oral health professionals’ support as well as the UoM.

### Variables

The outcome variable was the UoM. It was obtained through a “Yes/No” question “Did you use a mouthguard when playing basketball?”

There were several independent variables, including the level of knowledge, which was assessed using ten “Right or Wrong” questions regarding dental injury (definition, cause, type of injury, prevention related to basketball) and mouthguards (definition, type of mouthguards, advantages, and access to it). The right answer was given score 1 and the wrong answer was given score 0. From these answers, the total score for each participant is determined by adding up all the scores from the ten questions. This result was then categorized into three levels; namely, Low (1–5); Fair (6–7); Good (8–10).

The level of awareness (attitude towards use of mouthguards) was assessed using three statements with the five-point Likert scale from 1 to 5 (1: do not agree at all; 5: definitely agree). Three statements were: “*It is very important to prevent dental injuries while playing basketball*”; “*Athlete must wear mouthguards while practicing and playing basketball*” and “*There should be rules requiring use of mouthguard during basketball matches and practices*”. From these answers, the total score for each participant is determined by adding up all the scores from the three statements. Based on the total score, participants’ awareness levels were categorized into three: Low (3–8); Fair (9–11); Good (12–15).

Social support was composed of five items that were classified on a Likert scale of 1 to 5 (1: do not agree at all; 5: definitely agree), and it comprised two statements about parental support (“*My parents told me to use a mouthguard during basketball activities*” and “*My parents took me to the dentist for a consultation regarding the risks of dental trauma in sports*”), one statement about support from friends (“*My friends advised me to use a mouthguard during basketball activities*), one statement about support from coaches (“*My coach recommended me to use a mouthguard during basketball activities*”), and one statement about the organizing committee’s support (“*In basketball matches, the committee requires the use of a mouthguard*”). Each statement was categorized into two, namely a score of 1–3 as “without support” (value 0) and a score of 4 or 5 as “with support” (value 1). The scores from the five statements are then added up (minimum score 0 and maximum 5). The total results are grouped into two, namely less support (total score 0–2) and more support (total score 3–5).

Oral health professionals’ support was obtained through two statements regarding the involvement of dentists (“*Dentists have ever informed me about dental and orofacial injuries and their prevention*”) and the adequacy of the information (“*Oral health professionals, through various media such as posters, leaflets, the internet, etc.*, *have given me enough information regarding dental and orofacial injuries*”). OHPS also used the five-point Likert scale graduated from 1 to 5 (1: do not agree at all; 5: definitely agree). Each statement was categorized into two, namely a score of 1–3 as “without support” (value 0) and a score of 4 or 5 as “with support” (value 1). The scores from the two statements are then added up (minimum score 0 and maximum 2). The total results are grouped into two, namely less support (total score 0) and more support (total score 1–2).

Injury experience was assessed using a “Yes/No” question, “*Have you ever experienced on dental or orofacial injury*?”

Durations of basketball careers were measured using two questions, namely: “*How long have you been playing basketball (in years)?*” and “*How much time do you spend playing basketball? (hour(s)/week)?*”.

### Data analysis

Data were analyzed using Statistical Package for Social Science (SPSS) version 24.0 (IBM Chicago-ILL, USA). For statistical analyses, the level of significance was set at 5% (p < 0.05). The chi-square test (for categorical variables) was used to assess associations between the UoM and other variables. The independent-sample t-test was performed to assess differences in several continuous variables between outcome groups. Odds ratios (ORs) of the UoM were calculated for several variables using binary logistic regression. A model was also proposed with The Partial Least Square-Statistical Equation Model (PLS-SEM) [24] using WarpPLS 8.0 [25]. Model fit was evaluated with classic and additional indices.

## Results

The questionnaire’s validity was tested using 30 basketball players who were not involved in this study. The 21 items in the questions were all considered valid. Every item had r > 0.361. Since it reached 0.918 (> 0.60), Cronbach’s alpha was considered reliable.

In Table 1, there were 286 basketball players as the final number of participants; 127 of them were male and 159 were female. Of them, 86 (30.1%) used mouthguards and 87 (30.4%) had previous injury experiences. Among 87 players, the injury types were laceration (41.4%), fracture (12.6%), extrusion / intrusion (11.5%), dislocation of jaws (9.2%), avulsion (8.05%), don’t know (17.25%).

Due to only one participant scored in the “low” category (for knowledge and awareness), the respondent’s data was grouped into the “fair” category for further analysis. Age, length of basketball career, and amount of time spent playing each week all differed significantly between mouthguard users and non-users. The UoM was significantly associated with awareness level, injury experience, social support, and OHP support.

This result was also supported by a variety of variables’ ORs, which range from 1.28 to 5.97, as shown in Table 2. In term of probability to use mouthguards, female players were 1.3 times more likely than male players. Players from outside the Java Islands were 1.28 times more likely than its counterpart. For level of knowledge, players in “good” group were 1.47 times more likely than another group. For level of awareness, players in “good” group were 2.46 times more likely than “fair” group. Players with injury experience were 2.88 times more likely to use mouthguards than players without injury experience. Players with more social support were 5.97 times more likely to use mouthguards than players with less social support. Players who received more OHP support were 1.5 times more likely to use mouthguards than those who received less OHP support.

**Table 1:** Variables related to Use of Mouthguards among basketball players in Indonesia

Variables				Use of Mouthguards		*p-value*
				NO (n:200)	YES (n:86)	
				*Mean ± SD*	*Mean ± SD*	
Age (years)				19.98 (3.67)	21.29 (3.38)	0.005*
Height (cm)				166.67 (8.80)	164.74 (8.68)	0.083*
Weight (kg)				60.73 (13.99)	59.72 (14.11)	0.576*
Duration (in years)				5.55 (3.77)	4.55 (3.51)	0.036*
Playing Basketball (hours/week)				6.09 (5.31)	4.74 (3.91)	0.035*
Score of Knowledge				7.75 (2.59)	8.17 (2.44)	0.192*
			Total n:286	NO (n:200)	YES (n:86)	
				n(Column %)	n(Column %)	
Gender		Male	127 (44.4)	94 (47.0)	33 (38.4)	0.178**
		Female	159 (55.6)	106 (53.0)	53 (61.6)	
Residence		Java Islands	187 (65.4)	134 (67.0)	53 (61.6)	0.381**
		Outside Java	99 (34.6)	66 (33.0)	33 (38.4)	
Level of Knowledge		Low	1 (0.3)	1 (0.5)	0 (0)	0.789**
		Fair	25 (8.7)	17 (8.5)	8 (9.3)	
		Good	260 (91.0)	182 (91.0)	78 (90.7) ko	
Level of Awareness		Low	1 (0.3)	1 (0.5)	0 (0)	0.002**
		Fair	130 (45.5)	104 (52.0)	26 (30.2)	
		Good	155 (54.2)	95 (47.5)	60 (69.8)	
Experience of Injury		None	199 (69.6)	154 (77.0)	45 (52.3)	< 0.001**
		Have Injury	87 (30.4)	46 (23.0)	41 (47.7)	
Social Support		Less	167 (58.4)	145 (72.5)	22 (25.6)	< 0.001**
		More	119 (41.6)	55 (27.5)	64 (74.4)	
OHP Support		Less	155 (54.2)	129 (64.5)	26 (30.2)	< 0.001**
		More	131 (45.8)	71 (35.5)	60 (69.8)	

*obtained from T-Test ; **obtained from chi-square test; Bold denote significance < 0.05.

**Table 2:** Odd Ratios of Various Groups in the Use of Mouthguards among basketball players in Indonesia

Variables		OR	(95%CI)
Gender	Male	1	
	Female	1.30	(0.76–2.25)
Residence	Java Islands	1	
	Outside Java	1.28	(0.73–2.25)
Level of Knowledge	Fair	1	
	Good	1.47	(0.83–2.63)
Level of Awareness	Fair	1	
	Good	**2.46**	**(1.40–4.32)**
Experience of Injury	None	1	
	Have Injury	**2.88**	**(1.66–5.02)**
Social Support	Less	1	
	More	**5.97**	**(2.99–11.95)**
OHP Support	Less	1	
	More	1.53	(0.77–3.06)

Bold denotes significance < 0.05. *CI: Confidence Interval*

**Fig. 1:**
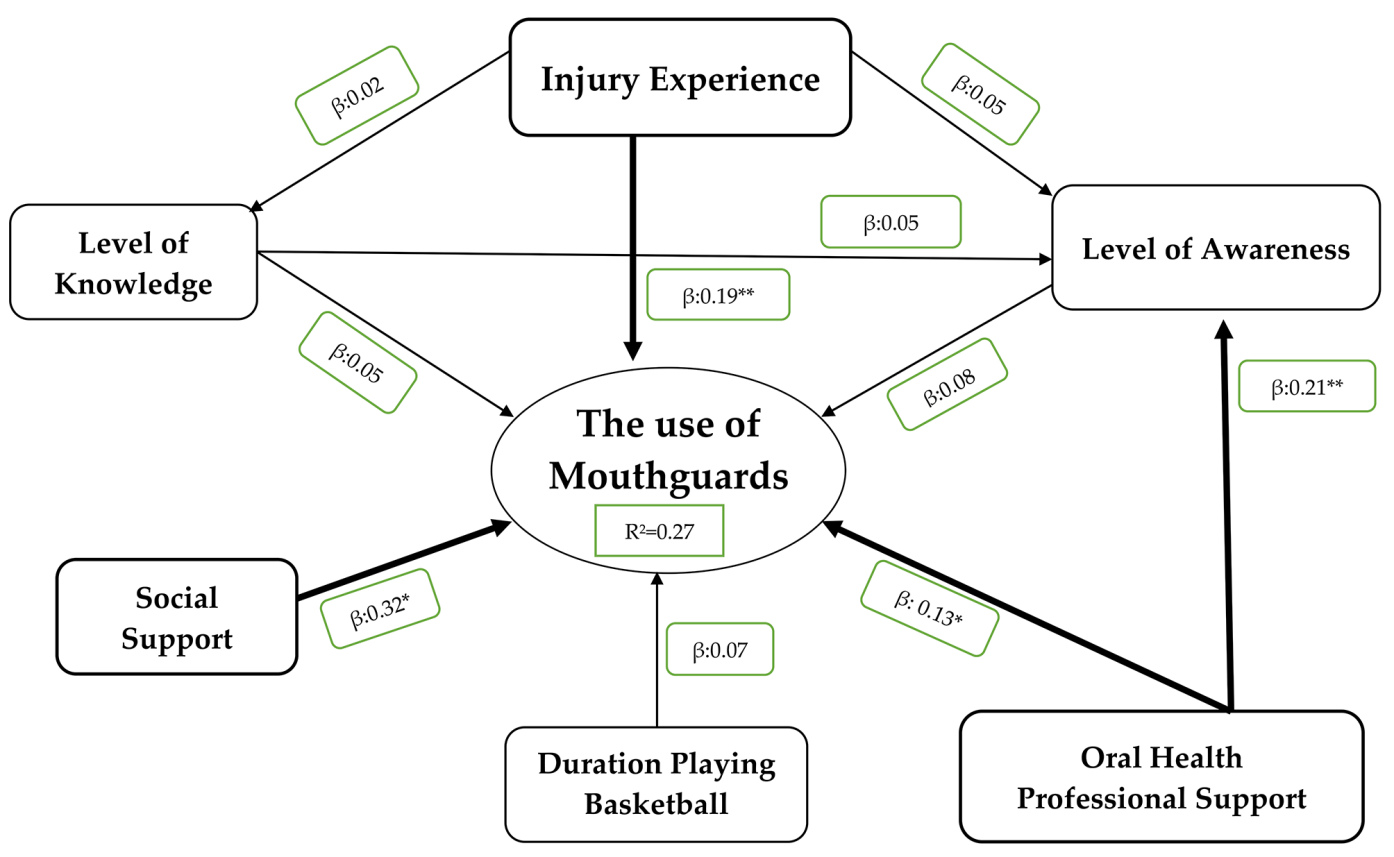
Path diagram factors related to the use of mouthguard among basketball players in Indonesia

The path model was evaluated between variables of interest such as the level of knowledge, level of awareness, injury experience, duration of basketball career, social support, and OHP support, as well as the UoM. PLS-SEM was performed, and Fig. 1 shows the path model among variables related to the UoM.

Utilizing both classic and additional indices, the path model’s fit was assessed. For the classic indices, the average path coefficient (APC) was 0.118 (p = 0.011); Average R-squared (ARS) was 0.107 (p = 0.017); Average adjusted R-squared (AARS) was 0.097 (p = 0.024); Average block VIF (AVIF) was 1.318 and Average full collinearity VIF (AFVIF) was 1.487 (the values of AVIF and AFVIF are regarded as ideal if ≤ 3.3); *Tenenhaus Goodness of Fit* (GoF) was 0.319 (the value was regarded as medium). For the additional indices, Simpson’s paradox ratio (SPR) was 0.900 (acceptable if ≥ 0.7, ideally = 1). R-squared contribution ratio (RSCR) was 0.995 (acceptable if ≥ 0.9, ideally = 1). Statistical suppression ratio (SSR) was 0.900 (acceptable if ≥ 0.7) and Nonlinear bivariate causality direction ratio (NLBCDR) was 1 (acceptable if ≥ 0.7). Every index reached the suggested standard for a good fit or an acceptable fit [25]. R2 was 0.27 (27%).

Figure 1 shows that for the direct effect of injury experience, social support and OHP support were significant; in other words, the direct effect of these factors supported UoM with β = 0.19, β = 0.32, and β = 0.13, respectively. Meanwhile, the level of knowledge, level of awareness, and duration of basketball career had insignificant support for UoM with β = 0.05, β = 0.08, and β = 0.07, respectively. The indirect effect between knowledge and UoM, as well as that between OHPS and UoM (which was mediated by the level of awareness), was also insignificant with β = 0.05 and β = 0.08, respectively. The indirect effect between injury experience to UoM which was mediated by knowledge and awareness, was also insignificant with β = 0.02 and β = 0.05, respectively. These latter findings were categorized as direct-only non-mediation to the relationship between injury experience to UoM.

## Discussion

In this study, 30.1% of basketball players used mouthguards while the rest (69.9%) did not use them. Although the proportion of UoM in this study was relatively higher than that in previous studies [23, 26], it was still considered low as similar findings among rugby players in Malaysia [27]. The low rate of UoM is a common phenomenon across countries. Even though basketball players usually have previous injury experiences, 52.9% of them still hesitate to use mouthguards. This study’s findings may strengthen the motivation of various stakeholders to promote the UoM among athletes, especially those at high risk of injury. There are still efforts that we should address regarding the UoM among such athletes.

According to the theory of behavior change [28, 29], the UoM by athletes is associated with predisposing factors (internal) such as knowledge, awareness, and experience (injury experience and career as basketball player), as well as external factors (enabling and/or reinforcing) such as social support and oral health professionals’ involvement. In this study, it was revealed that almost all factors were associated significantly with UoM except the LoK. Hence, the first null hypothesis that the level of knowledge (LoK), injury experience, awareness, duration of basketball career, support from the social environment and oral health professionals, were identified and associated significantly with the UoM among basketball players in Indonesia, was accepted. The level of knowledge did not have a statistically significant association with UoM. However, the average score of the user group is higher than that of the non-user group, despite the fact that the difference in knowledge between the two groups is not statistically significant, indicating that knowledge plays a role in the UoM among Indonesian basketball players. A non-significant probability of almost 1.5 times is indicated by adequate knowledge. According to a study in Saudi Arabia, sports participants with good knowledge were nearly four times more likely to use mouthguards than those with less knowledge [30]. Several sound behavior theories connect knowledge to practice or action. Because of the high level of injury in this sport, dental emergency literacy could be an interesting topic, given that this still requires attention among non-dental students in Indonesia [21]. Even with good awareness, a considerable probability of roughly 2.5 times is apparent. Athletes are motivated to wear mouthguards by the protective benefits of the latter and their personal history of dental injuries [31]. The proportion of participants with a good level of awareness (54.19%) is similar to that reported in a previous study conducted in Greece but lower than that reported in the USA [32], implying that findings regarding the level of awareness of UoM continue to vary, despite visible similarities in developing countries and differences in developed countries. This finding supports previous findings that, while athletes’ knowledge and awareness of mouthguards is good or even high, their use is nevertheless low. This can be assumed that the level of knowledge and awareness may not always correspond to the action of using a mouthguard. Although the mechanism of “from knowledge to action” is not always linear as revealed in Fig. 1 (that knowledge and awareness have less effect on mouthguard use), which means that knowledge does not always become an action, this does not diminish the role of knowledge in action or practice [33]. Oral health education and/or promotion initiatives that are integrated with multiple factors and other connected parties (such as the proposed model) are predicted to be one option.

For the injury experience, the probability is significant (almost three-fold with logistic regression) and direct effect is also significant (with SEM). A previous study reported that athletes having a history of oral injuries were considerably more likely to use mouthguards than players without such a history [34]. Another previous study stated that sport participants with dental injuries experience had probability 1.4 times to use mouthguards compare to sports participants without such experience [30]. According to the COM-B (Capability-Opportunity-Motivation-Behavior) theory [35], injury experience as an unpleasant experience may naturally motivate athletes to avoid it in the future. At the same time, this is a great opportunity for OHP to educate (increase capability) basketball players and promote the use of mouthguards (behavior).

A probability of 1.5 times is also provided by the support from the OHP. A previous study stated that dentists play an important role in developing strategies to encourage athletes to use mouthguards. Dentists should be required to participate effectively in sports clubs in order to educate federations, coaches, and athletes about the importance of mouthguards [8]. According to the integrated behavior model, to engage in certain behaviors, everyone generally needs knowledge and skills [36]. An OHP is one of the qualified parties that may provide athletes with proper education regarding the UoM. This recommendation is also reinforced by a prior study that suggested that players, their coaches, and their caretakers should receive continual education about the importance of mouthguards and the management of traumatic dental injuries (TDIs) [37]. Nearly six times the probability is provided by social support, which, in this study, encompasses parents, friends, and coaches as well as committees. Social support plays an important role in determining a person’s behavior and/or health condition. Social support, which can manifest as close relationships and care, is undoubtedly related to health and well-being at all stages of life [38]. Aside from the behavior theory, this study suggested two thriving components, social well-being, and physical well-being [38], which are that are connected in a proposed model. The proposed model provided a coefficient of determination of 27%. This implies that the six variables (level of knowledge, level of awareness, injury experience, duration practicing, social support, and OHP support) could explain 27% of the variance in the UoM. This coefficient of determination is considered high, especially for behavioral research [39]. Hence, the second null hypothesis that these factors could be directly or indirectly connected significantly or insignificantly to construct a model with a high coefficient of determinants, was accepted. Variations in the use of mouthguards were explained by simultaneous interactions between several factors, including both statistically significant and nonsignificant ones. Injury prevention efforts should take into account every aspect as thoroughly as feasible. Moving forward from this suggested model, which highlights the importance of social support and OHP support, we might be able to implement oral health education initiatives based on the players’ surroundings, such as the dentists’ socializing of mouthguards at the basketball arena prior to tip-off. Parents, coaches, and friends who might serve as role models for mouthguard use at the time of this socialization may be more effective. This activity could be a complement to the basketball committee’s or association’s rules requiring players to wear mouthguards. A previous study in Japan found that such regulations could effectively increase the use of mouthguards [40]. As stated in a proposed social dentistry framework by Bedos *at el* [41], this effort is a health effort that involves three imbricated levels, namely individual and family (micro-level), friends and coaches (meso-level), and committee and basketball association (macro-level). The framework depicts these three levels separately; however, the proposed model allows all three levels to participate in a single oral health education effort at the same time. This is possibly one of the model’s strengths, in addition to the high coefficient of determination.

The overall findings of this study must be interpreted in light of its limitations. First, the cross-sectional study design naturally cannot confirm the direction of the causality relationship. However, the arrows in PLS-SEM are always single headed, representing directional relationships. Single-headed arrows are considered predictive relationships and could be interpreted as causal relationships with strong theoretical support [24]. Second, the convenience sampling design -as one of non-probability- does not allow us to generalize the findings. Third, the limitations of collecting data online, such as the difficulty in determining whether the participants completed the questionnaire on their own or not. Another challenge is determining whether the same participant completed the questionnaire only once or several times. To avoid this, a full name and an email address are required to fill out the Google form. Despite its limitations, this study can be considered the first piece of investigation on the use of mouthguards among basketball players in Indonesia as well as the first study proposed a model using SEM analysis, and the SEM model which was encompassed several factors simultaneously, may be applicable in other countries and various sports.

## Conclusions

The UoM among basketball players in Indonesia was related to several factors. Level of awareness (OR = 2.46), injury experiences (OR = 2.88), social support (OR = 5.97), and oral health professionals’ support (OR = 1.53) had important roles to play. Nonetheless, other factors such as knowledge (OR = 1.47), length of basketball career and duration of practicing basketball must be considered to increase the UoM among these athletes. These factors were constructed to propose a model that could explain 27% of the relationships between variables and the UoM among Indonesian basketball players. It is expected that future research will look into other factors such as the professional level of the athletes that may affect the UoM by basketball players in Indonesia, complementing and expanding on the proposed model.

## Data Availability

The data presented in this study are available on request from the corresponding author. The data are not publicly available due to ethical and privacy policy.

## Abbreviations

UoM: Use of mouthguards
SPSS: Statistical Package for Social Science
SEM: Structural equation modelling
OHP: Oral health professional
OHPS: Oral health professional support
LoK: Level of Knowledge
PLS-SEM: The Partial Least Square-Statistical Equation Model

## References

[1] Stamos A, Mills S, Malliaropoulos N, Cantamessa S, Dartevelle JL, Gündüz E, Laubmeier J, Hoy J, Kakavas G, Le Garrec S (2020). The European Association for Sports Dentistry, Academy for Sports Dentistry, European College of Sports and Exercise Physicians consensus statement on sports dentistry integration in sports medicine. Dent Traumatol.

[2] Petti S, Glendor U, Andersson L (2018). World traumatic dental injury prevalence and incidence, a meta-analysis—one billion living people have had traumatic dental injuries. Dent Traumatol.

[3] Young EJ, Macias CR, Stephens L (2015). Common dental injury management in athletes. Sports Health.

[4] Azodo CC, Odai CD, Osazuwa-Peters N, Obuekwe ON (2011). A survey of orofacial injuries among basketball players. Int Dent J.

[5] Castaldi C (1989). Sports dentistry. ASDC J Dent Child.

[6] Fernandes LM, Neto JCL, Lima TF, Magno MB, Santiago BM, Cavalcanti YW (2019). De Almeida LdFD: the use of mouthguards and prevalence of dento-alveolar trauma among athletes: a systematic review and meta‐analysis. Dent Traumatol.

[7] Green JI (2017). The role of mouthguards in preventing and reducing sports-related trauma. Prim Dent J.

[8] Meyfarth SRS, Rodrigues KAB, Von Held R, Sarkis P, Gouvea Junior LEC, Antunes LAA, Antunes LS. An analysis of athletes’ knowledge, acceptance and usability toward custom-made mouthguards: uncontrolled before–after study. Sport Sci Health 2022.

[9] Parker K, Marlow B, Patel N, Gill D (2017). A review of mouthguards: effectiveness, types, characteristics and indications for use. Br Dent J.

[10] Lloyd JD, Nakamura WS, Maeda Y, Takeda T, Leesungbok R, Lazarchik D, Dorney B, Gonda T, Nakajima K, Yasui T (2017). Mouthguards and their use in sports: report of the 1st International Sports Dentistry Workshop, 2016. Dent Traumatol.

[11] Gawlak D, Mańka-Malara K, Kamiński T, Łuniewska M, Mierzwińska‐Nastalska E (2016). Comparative evaluation of custom and standard boil and bite (self‐adapted) mouthguards and their effect on the functioning of the oral cavity. Dent Traumatol.

[12] Cornwell H, Messer LB, Speed H (2003). Use of mouthguards by basketball players in Victoria, Australia. Dent Traumatol.

[13] Bergman L, Milardović Ortolan S, Žarković D, Viskić J, Jokić D, Mehulić K (2017). Prevalence of dental trauma and use of mouthguards in professional handball players. Dent Traumatol.

[14] Prieto-González P, Martínez-Castillo JL, Fernández-Galván LM, Casado A, Soporki S, Sánchez-Infante J (2021). Epidemiology of sports-related injuries and associated risk factors in adolescent athletes: an injury surveillance. Int J Environ Res Public Health.

[15] Tuna EB, Ozel E (2014). Factors affecting sports-related orofacial injuries and the importance of mouthguards. Sports Med.

[16] Ma W (2008). Basketball players’ experience of dental injury and awareness about mouthguard in China. Dent Traumatol.

[17] Committee AAoPDCA, Affairs AAoPDCoC (2005). Policy on prevention of sports-related orofacial injuries. Pediatr Dent.

[18] Jenis Cabang Olahraga yang Populer di Indonesia., Apa Saja? [https://sports.okezone.com/read/2021/01/28/43/2352522/jenis-cabang-olahraga-yang-populer-di-indonesia-apa-saja?page=1].

[19] Association WM (2013). World Medical Association Declaration of Helsinki: ethical principles for medical research involving human subjects. JAMA.

[20] Harriss DJ, MacSween A, Atkinson G (2019). Ethical Standards in Sport and Exercise Science Research: 2020 update. Int J Sports Med.

[21] Novrinda H, Darwita RR, Subagyo KA. The Effect of Educational Video on COVID-19 and Dental Emergency literacy among students during pandemic era. Eur J Dentistry 2022. 10.1055/s-0042-1743152 PMC9949924 35436791

[22] Mustafa RM, Alrabadi NN, Alshali RZ, Khader YS, Ahmad DM (2020). Knowledge, attitude, Behavior, and stress related to COVID-19 among Undergraduate Health Care students in Jordan. Eur J Dentistry.

[23] Akodu A, Akinbo S, Ajiboye A (2017). Injury patterns and perceived risk factors among basketball players in Nigeria. Med Sportiva: J Romanian Sports Med Soc.

[24] Hair JF Jr, Hult GTM, Ringle CM, Sarstedt M, Danks NP, Ray S. Partial least squares structural equation modeling (PLS-SEM) using R: a workbook. Springer Nature; 2021.

[25] Kock N (2022). WarpPLS user Manual: Version 8.0. In. Loredo.

[26] Collins CL, McKenzie LB, Roberts KJ, Fields SK, Comstock RD (2015). Mouthguard BITES (behavior, impulsivity, theory evaluation study): what drives mouthguard use among high school basketball and baseball/softball athletes. J Prim Prev.

[27] Liew AKC, Abdullah D, Wan Noorina WA, Khoo S (2014). Factors associated with mouthguard use and discontinuation among rugby players in M alaysia. Dent Traumatol.

[28] Green LW, Ottoson JM, García C, Hiatt RA, Roditis ML (2014). Diffusion theory and knowledge dissemination, utilization and integration. Front Public Health Serv Syst Res.

[29] Green LW (1984). Modifying and developing health behavior. Annu Rev Public Health.

[30] Al-Arfaj I, Al-Shammari A, Al-Subai T, Al-Absi G, AlJaffari M, Al-Kadi A, El Tantawi M, Al-Ansari A (2016). The knowledge, attitude and practices of male sports participants to sports-related dental trauma in Khobar and Dammam, Saudi Arabia–A pilot survey. Saudi Dent J.

[31] Petterson C, Van Wichen A, Antoun J. Sports mouthguards: a review. NZ Dent J 2020, 116(1).

[32] Elena-Lito E, Ioannis K, Nikolaos K. Use of mouthguards by amateur basketball athletes in Greece and the USA. Trauma Cases and Reviews 2019, 5(1).

[33] West S, van Kerkhoff L, Wagenaar H (2019). Beyond linking knowledge and action: towards a practice-based approach to transdisciplinary sustainability interventions. Policy Stud.

[34] Tiryaki M, Saygi G, Yildiz SO, Yildirim Z, Erdemir U, Yucel T (2017). Prevalence of dental injuries and awareness regarding mouthguards among basketball players and coaches. J Sports Med Phys Fitness.

[35] Michie S, van Stralen MM, West R (2011). The behaviour change wheel: a new method for characterising and designing behaviour change interventions. Implement Sci.

[36] Montano DE, Kasprzyk D (2015). Theory of reasoned action, theory of planned behavior, and the integrated behavioral model. Health Behavior: Theory Research and Practice.

[37] Kroon J, Cox JA, Knight JE, Nevins PN, Kong WW (2016). Mouthguard use and awareness of junior rugby league players in the Gold Coast, Australia: a need for more education. Clin J Sport Med.

[38] Feeney BC, Collins NL (2015). A new look at social support: a theoretical perspective on thriving through relationships. Personality and Social Psychology Review.

[39] Hair JF Jr, Hult GTM, Ringle C, Sarstedt M. A primer on partial least squares structural equation modeling (PLS-SEM). In., 2nd edition edn. Los Angeles: Sage publications; 2017.

[40] Hayashi K, Churei H, Tanabe G, Togawa K, Chowdhury RU, Ueno T (2020). Improving the wearing rate of Mouthguards in the Youth Rugby Category affects the total future mouthguard wearing rate. Dentistry J.

[41] Bedos C, Apelian N, Vergnes JN (2018). Social dentistry: an old heritage for a new professional approach. Br Dent J.

